# Barcoding of Ancient Lake Ostracods (Crustacea) Reveals Cryptic Speciation with Extremely Low Distances

**DOI:** 10.1371/journal.pone.0121133

**Published:** 2015-03-26

**Authors:** Ivana Karanovic

**Affiliations:** 1 Department of Life Science, Hanyang University, Seoul, South Korea; 2 Institute for Marine and Antarctic Studies, University of Tasmania, Hobart, Tasmania, Australia; The New York Botanical Garden, UNITED STATES

## Abstract

Ostracods are drastically reduced crustaceans, with never more than eight appendages enclosed between two valves, leaving only a limited number of morphological characters for species delineation. Conservative morphology of characters used to define genera, along with high variability of characters used to define species are creating problems in applying a morphospecies concept. A high intraspecific variability in a Lake Biwa (Japan) endemic, *Physocypria biwaensis* (Okubo, 1990), has been observed previously but was never studied in detail. Two sympatric forms, differing in pigmentation and size, suggest a presence of reproductive isolation. The aim of this study is to employ molecular and morphometric tools to aid in species delineation within *P*. *biwaensis* complex and reconstruct their phylogenetic relationships. A fragment of the mtCOI gene was amplified from 30 specimens, and an additional 37 specimens were studied for morphological characters. Resulting phylogenies showed that each morphologically distinct form is associated with a distinct phylogenetic group based on mtDNA. The average pairwise distance is very low (5%), indicating a recent divergence time. I speculate that there is a possibility that one of them originated in the lake, while the other probably colonized it afterwards. This seems to be supported with an apparent niche partitioning at different depths. In spite of the fact that traditionally used sexual characters are highly variable in these two species, the morphometric analysis of shell and soft part related characters clearly delineates them and suggests that such characters may be useful for future detection of seemingly cryptic ostracod species.

## Introduction

Development of molecular tools for species delineation has considerably increased the number of cryptic species worldwide. It has been shown [1] that they are almost evenly distributed among major metazoan taxa and biogeographic regions, which has theoretical and practical consequences for many spheres of biology, but in particular for the global conservation efforts and taxonomic initiatives. In the past decade several ostracod species from different lineages have been studied in the light of possible cryptic speciation [2, 3, 4, 5, 6], but rarely have these studies resulted in a clear morphological delineation of species and/or eventual description of the new ones (see [4, 7]). In certain cosmopolitan ostracods close to 40 cryptic species have been suggested from Europe and North Africa [2], some of which were identified as recent invaders of the Australian freshwater from Europe [3], but none morphologically defined.

The level of molecular divergence in the barcoding mtCOI sequences between species largely varies depending on the animal group or even family within certain groups. Léfebure *et al* [8] measured *COI* divergence rates across a range of crustacean taxa and concluded that 16% patristic distance between two *COI* sequences is the threshold for species delineation in this phylum. On the other hand, in some studies of branchiopod crustaceans a 3% divergence threshold was used to delineate potential species [9], which is similar to some groups of butterflies [10]. Da Silva *et al*. [11] showed that the mean sequence distances between species in one genus vary greatly across decapod families, ranging from around 6% to 20%. Sometimes divergence rates as high as 12% fail to reveal any morphological difference in crustaceans (see [12]). In ostracods, the *COI* divergence rates between well-defined morphological species of one genus vary from 6% to 14% [7], while apparently no morphological differences have been found in some cosmopolitan species where the average pairwise distances between geographically isolated populations can be as high as 20% [2].

High morphological similarity between genetically distinct populations often requires statistical and evolutionary tests to verify the existence of a significant difference and presence of cryptic species. Such methods include Automatic Barcode Gap Discovery [13]; the generalized mixed Yule coalescent model [14]; and the K/θ method [15]. Although these tests have great theoretical value and can be useful in large scale sequencing of certain species or regions, they have little application in taxonomic studies and practical species recognition where two or more closely related species live sympatrically. On the other hand, problems in applying the morphospecies concept are often associated with high intraspecific variability. In ostracod crustaceans, the number of morphological characters that can be used for species identification is limited by the extremely reduced body, which never contains more than eight appendages enclosed between two valves. Characters that are used to define genera (number of segments on the first antenna and thoracopods, chaetotaxy of the antenna and thoracopods, sexual characters, etc.) tend to be very conservative [16, 17]. On the other hand, there is often high intraspecific variability in the shell morphology. The chemo-physical properties of the shell mirror the environment where the animal lives, and are thus as plastic as the tolerance of the species to certain environmental conditions [18, 19]. In many cases, however, an array of different morphological forms can be found living in sympatry.

Smith & Janz [20] reported that the Lake Biwa (Japan) endemic ostracod, *Physocypria biwaensis*, has several forms that vary in the carapace shape and patterns of color patches, but they were not able to confidently divide the specimens into morphologically discrete species due to the alleged presence of a range of intermediate morphotypes. In addition, they found that sexual characteristics did not show any differences to warrant separation of samples into discrete taxa. However, they did not rule out the possibility that there may be two or more cryptic species in the lake.

Lake Biwa has a tectonic origin with the geological history dating back to approximately four million years, during which time it went through size and environmental changes, with the present conditions forming about 430,000 years ago [21]. As such, it has global significance for the study of the dynamic aspects of evolution [22]. Ancient lakes have been noted as hotspots of ostracod diversity and appear to hold up to 25% of the total number of freshwater species [23]. Forty ostracod species have been reported so far from Lake Biwa, of which 16 are known only from the lake, although the latter may be due to a lack of study of the surrounding area [24]. The shallow depths of Lake Biwa support the highest ostracod diversity, which rapidly decreases below the summer thermocline (developing at approximately 10–20 m depth, see [24]). Only three species are found in the deepest parts of the lake, i.e. below 50 m: *Fabaeformiscandona nishinoae* Smith & Janz, 2008, *Physocypria biwaensis*, and *Cytherissa lacustris* (Sars, 1863) (see [20]). The former two appear to be endemic, while the latter has a Holarctic distribution [16].

The aim of this study was to test if different morphotypes of *P*. *biwaensis* represent cryptic species using mitochondrial *COI* sequence data, and to find potentially useful morphological characters for their delineation. Mitochondrial *COI* sequences were also used to estimate divergence rates between the different forms, and different soft parts and shell characters were used for morphometric analyses.

## Material and Methods

### Sampling

**Fig 1:**
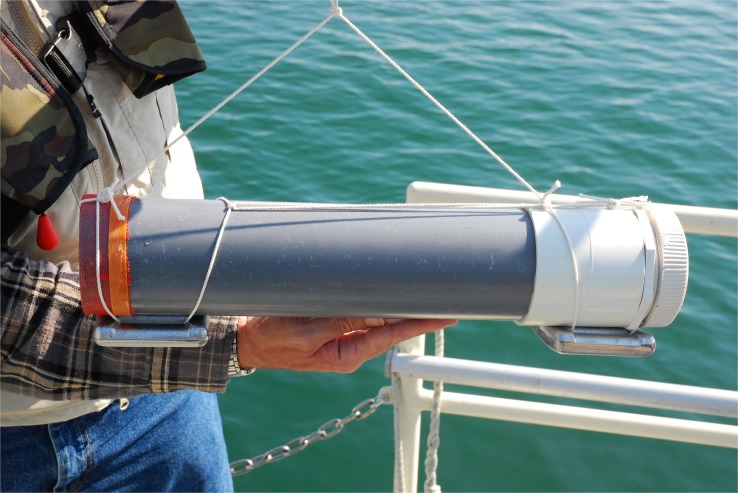
Collecting from Lake Biwa. Light traps used for collecting samples from deep parts of the lake (Photo R. Smith, Lake Biwa Museum).

Samples were collected from Lake Biwa (Japan) at the following three stations:

1. Littoral, close to the Lake Biwa Museum, 0.5–1 m depth, 35°04’31.96”N 135°54’04.79”E, 03 November 2013; collector T. Karanovic;
2. Buoy trap station 1, at 41 m depth, off Kitakomatsu, 35°14’41”N 135°58’20”E, 29 October 2013, collectors T. Karanovic and M. Grygier;
3. Buoy trap station 2, at 79.5 m depth, off Ohmi-Maiko, 35°13’13”N 135°58’ 39”E, 29 October 2013 (Fig. 1), collectors T. Karanovic and M. Grygier.

No specific permissions were required for these locations at Lake Biwa, and the field studies did not involve endangered or protected species.

The samples from the first station were collected using a plankton hand net (50μm mesh size), and immediately fixed in 95% ethyl alcohol. Specially constructed light traps (Fig. 1) were used to collect samples from the other two stations. They had a coarse net (2mm mesh size) covering the entrance to prevent small fish and other larger animals from entering. At each station, the trap was baited with a peace of fish, and an LED pocket-light was also inserted. The trap was then submerged into a crate filled with filtered lake water, and lowered to the lake bottom. The traps were left overnight connected to the solar powered blinking buoy and collected the following day. The trapped animals were fixed in 95% ethyl alcohol. All collected samples were sorted at the Lake Biwa Museum under a dissecting microscope, and the animals were transferred into 1.5 ml plastic vials in 95% ethyl alcohol.

### Taxonomy and morphometry

**Fig 2:**
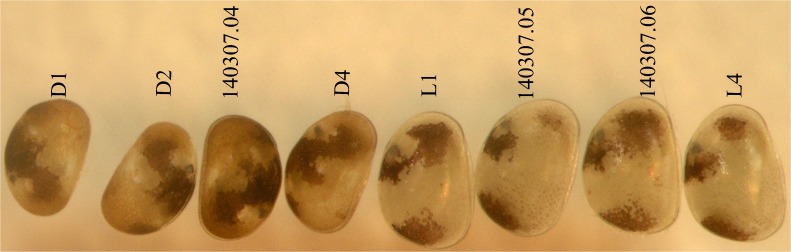
Light microscope photograph of *Physocypria biwaensis* collected from Lake Biwa at 41 m. Dark form, first four from left to right; light form, last four specimens. Long numbers above specimens correspond to *COI* sequences (see Fig. 10), while letters with numbers include specimens that were either measured, used for SEM, or just examined.

**Fig 3:**
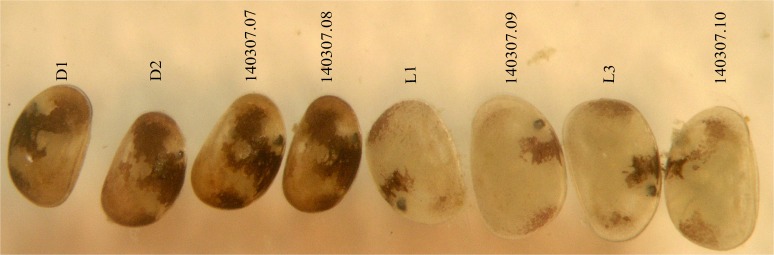
Light microscope photograph of *Physocypria biwaensis* (Okubo, 1990) collected from Lake Biwa at 79.5 m. Dark form, first four from left to right; light form, last four specimens. Numbers above specimens correspond to *COI* sequences (see Fig. 10), while letters with numbers indicate specimens that were either measured, used for SEM or just examined.

**Fig 4:**
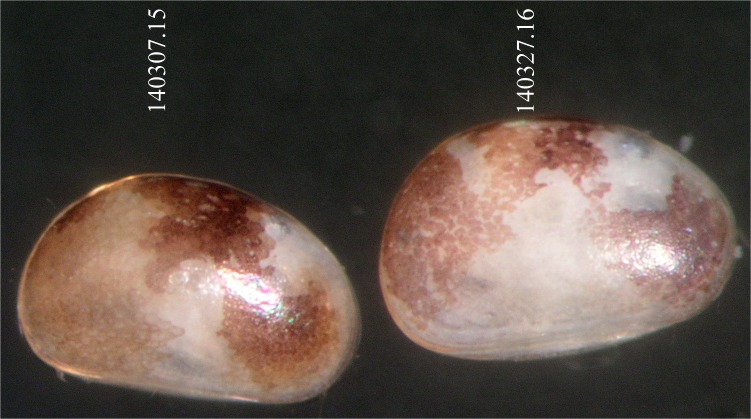
Light microscope photograph of *Physocypria biwaensis* (Okubo, 1990) collected from Lake Biwa at 79.5 m showing intermediate forms. Left, dark intermediate (DI); right, light intermediate (LI). Numbers above specimens correspond to *COI* sequences (see Fig. 10).

**Fig 5:**
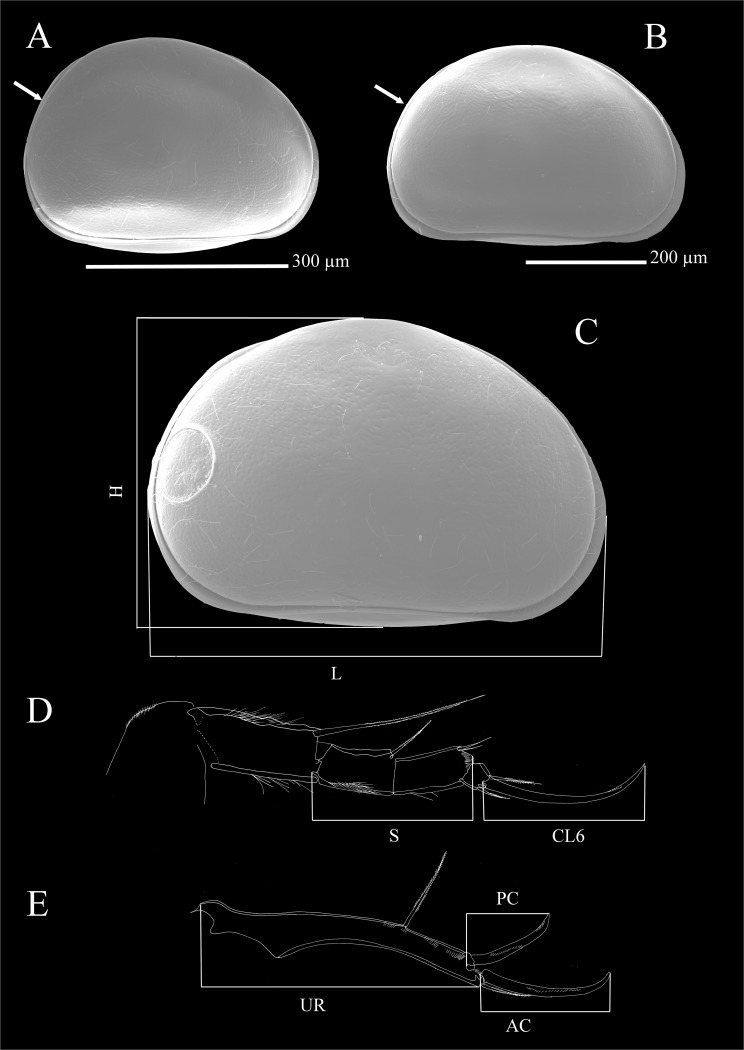
SEM photos (A, B, C) and line drawings (D, E) of *Physocypria biwaensis* showing morphological differences in the shell shape and morphometric measurements taken. A, female light form; B, female dark form; C, male dark form, measurements of the shell; D, male, dark form, measurements of the walking leg; E, male, dark form, measurements of the uropodal ramus. Arrows indicate the difference in the shape of the posterior margin. H, height; L, length; S, length of two segments; CL6, length of the terminal claw; CR, length of the uropodal ramus; AC, length of the anterior claw; PC, length of the posterior claw.

Two species belonging to *Physocypria* Vávra, 1897 were collected: *P*. *nipponica* Okubo, 1990 and *P*. *biwaensis* (Okubo, 1990). The former was collected from the first sampling station only, while the latter species was collected from trap stations (Table 1). They were identified based on their original descriptions [25] and subsequent short re-descriptions [20]. Two forms of *P*. *biwaensis* were present in both deep lake samples: a light form, with dark patches not forming continuous layers on the shell and with an overall whitish glow; and a dark form, which has more surface area covered with dark patches, that are more continuous and the specimens have an overall darker appearance (Figs. 2and 3). In addition, a couple of representatives which have an intermediate appearance (Fig. 4) were also present in the samples. In order to study their morphology I have dissected 19 specimens of the light and 18 specimens of the dark form. First, the measurements of the shell, such as length and height (Fig. 5C) were taken, after which the valves were removed. In many cases the shell was broken during handling and was not kept, while whenever valves were removed intact they were stored on SEM stubs or in alcohol. The soft parts were dissected with sharp tungsten entomological needles on a glass slide in a drop of CMC-10 mounting media (Masters Company, Inc., USA). For each dissected specimen the following dimensions were recorded: total length of the second and third segment of the walking leg, total length of the terminal claw on the same appendage (Fig. 5D), total length of the ramus on the uropodal ramus, and total length of the posterior and anterior claws on the same appendage (Fig. 5E). Each dissected and examined specimen was labelled according to its form (light or dark), depth from which it was collected (41 or 79 m), sex (male or female), and an additional number or letter.

**Table 1:** Number of collected *Physocypria* specimens and population structure at three sampling stations at Lake Biwa.

	0–1 m			41 m depth			79 m depth		
	Male	female	juvenile	male	female	juvenile	male	female	juvenile
*P*. *biwaensis* L	-	-	-	10	23	-	156	357	-
*P*. *biwaensis* D	-	-	-	21	13	20	12	6	-
*P*. *nipponica*	6	15	4	-	-	-	-	-	-

Specimens were dissected and photographed with an Olympus SZX12 dissecting microscope, equipped with an Olympus C-5050 digital camera. Soft parts were examined and measured with a Leica DM 2500 compound microscope, equipped with NPlan objectives and a drawing tube attachment.

Scanning Electron Micrographs (SEM) were taken with a Hitachi S-4700 scanning electron microscope at Eulji University (Seoul).

Morphometric measurements for statistical calculations are deposited on DRYAD (10.5061/dryad.1s6q2) and include data files: Appendix 1-Dryad, Appendix 2-Dryad.

### DNA extraction and amplification

A total of 45 specimens were used for DNA extraction and amplification: nine *P*. *nipponica*, 17 *P*. *biwaensis* light form (seven from 41 m and 10 from 79 m), 17 *P*. *biwaensis* dark form (seven from 41 m and 10 from 79 m), and two specimens identified as intermediate forms both from 79 m. Before DNA extraction some of the specimens were photographed (Figs. 2–4). I used whole body (soft parts and shell) for the DNA extraction. In the first step of the DNA extraction specimens were kept for 2–3 hours in distilled water. LaboPass Tissue Mini extraction kit (Cosmo Genetech Co., LTD, Korea) was used in all further steps of extraction, following the manufacturer’s protocol. Fragments of mitochondrial *COI* (on average 640 bp) were amplified using Folmer primers [26], using PCR method in the TaKaRa PCR Thermal Cycler Dice in 25 *μl* volumes, containing: 5 *μ*l of DNA template, 1x ExTaq Buffer, 1.25 units of TaKaRa Ex Taq, 0.25 mM of dNTP, 1 *μ*l each primer (10 *μ*M). The PCR protocol for *COI* fragment amplification consisted of initial denaturation for 5 minutes at 94°C, 40 cycles of denaturation for 1 minute at 94°C, annealing for 2 minutes at 46°C, extension for 3 minutes at 72°C. Final extension was at 72°C for 10 minutes. The PCR products were purified for sequencing reactions, using the LaboPass PCR Purification Kit (Cosmo Genetech Co., LTD, Korea) following the guidelines provided with the kit. DNA was sequenced on an ABI automatic capillary sequencer (Macrogen, Seoul, South Korea) using the same set of primers. Of 45 specimens, the PCR amplification was successful in 35 cases.

### Data analysis

**Fig 6:**
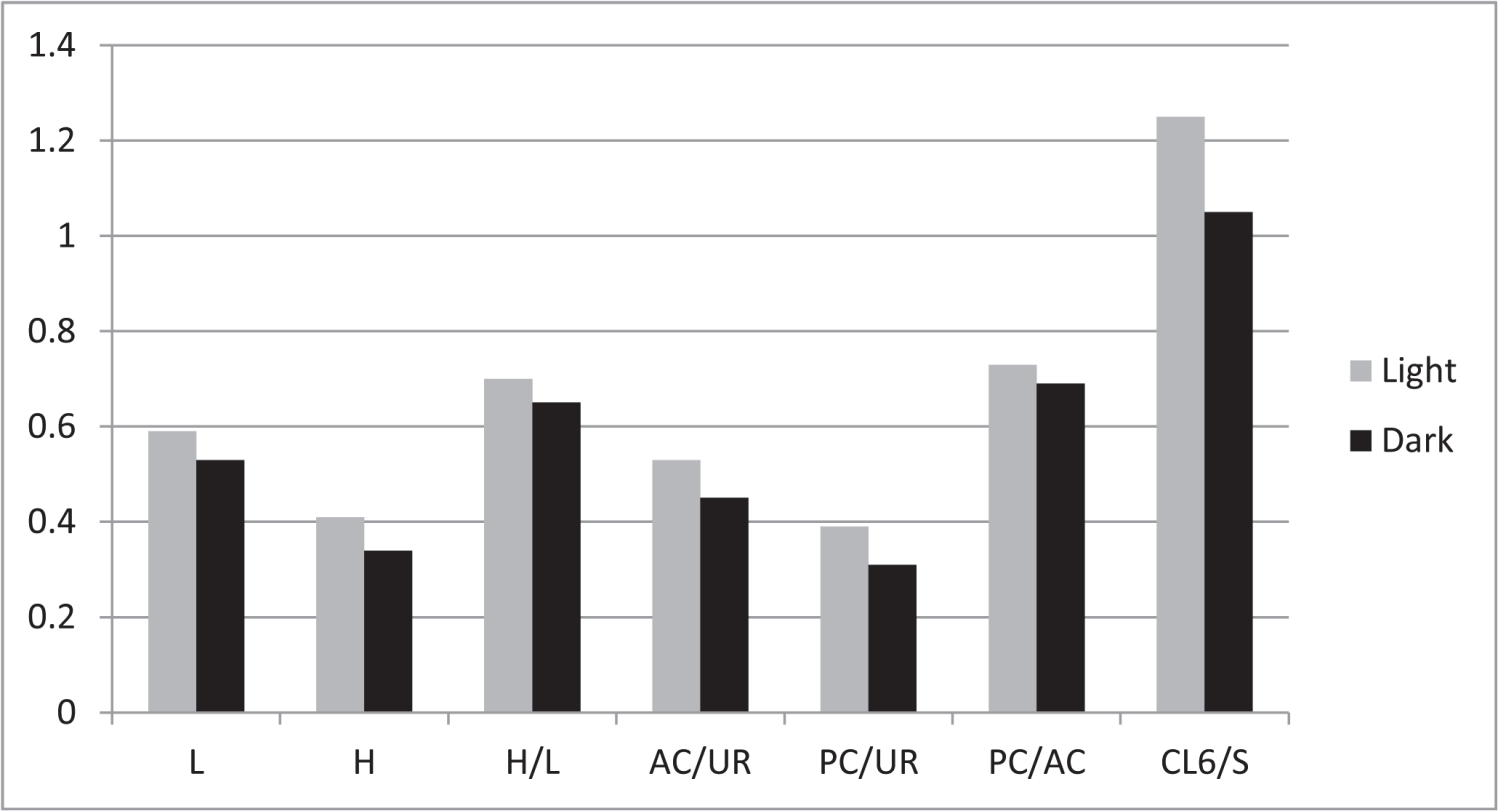
Histogram showing differences in average values of the morphometric data in two forms of *Physocypria biwaensis*. For morphometric abbreviations see Fig. 5.

**Fig 7:**
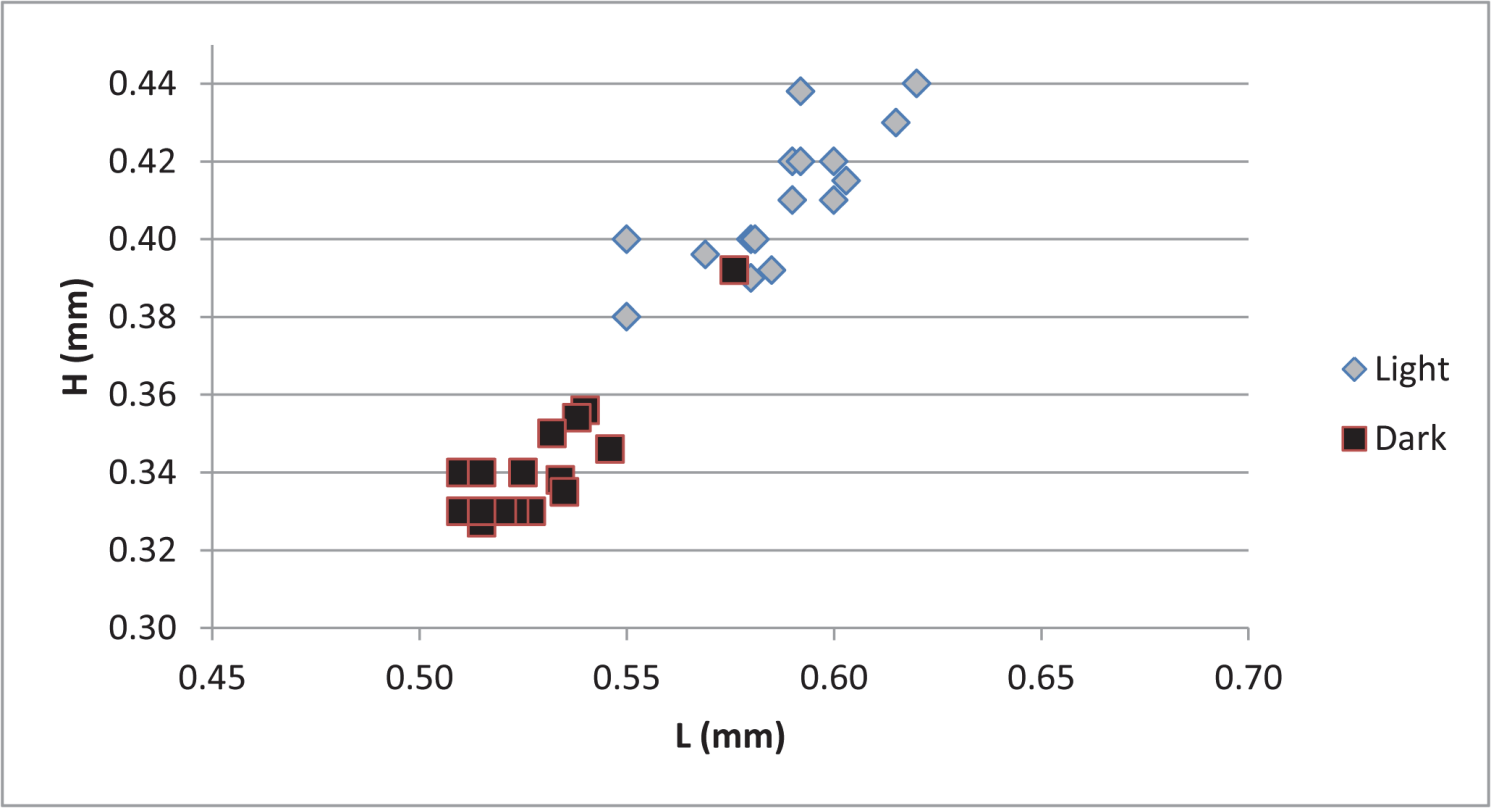
Scatter plot of length (L) and height (H) of the shell in two forms of *Physocypria biwaensis*.

**Fig 8:**
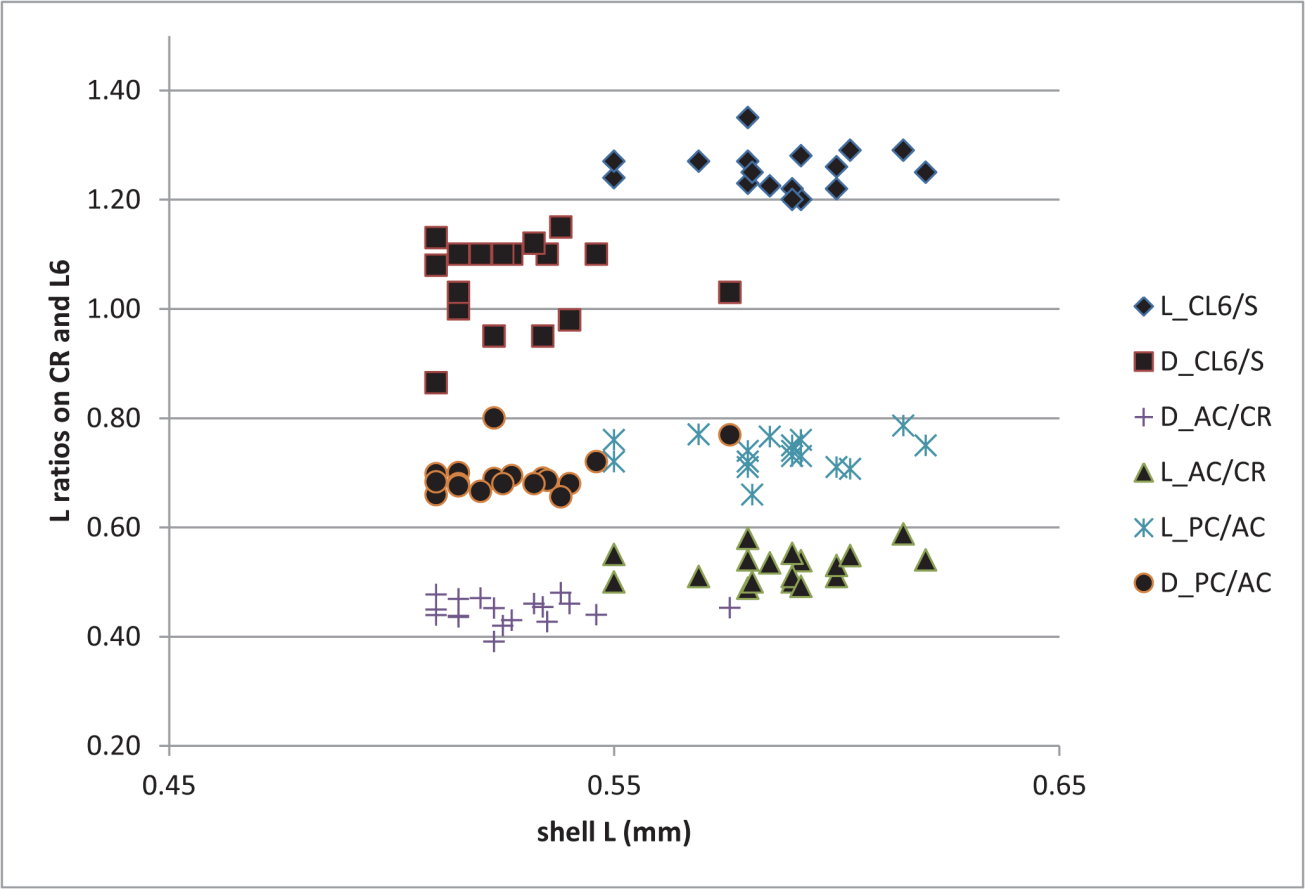
Scatter plot of all morphometric data measured on soft parts as a function of the shell length in two forms of *Physocypria biwaensis*. For morphometric abbreviations see Fig. 5.

**Fig 9:**
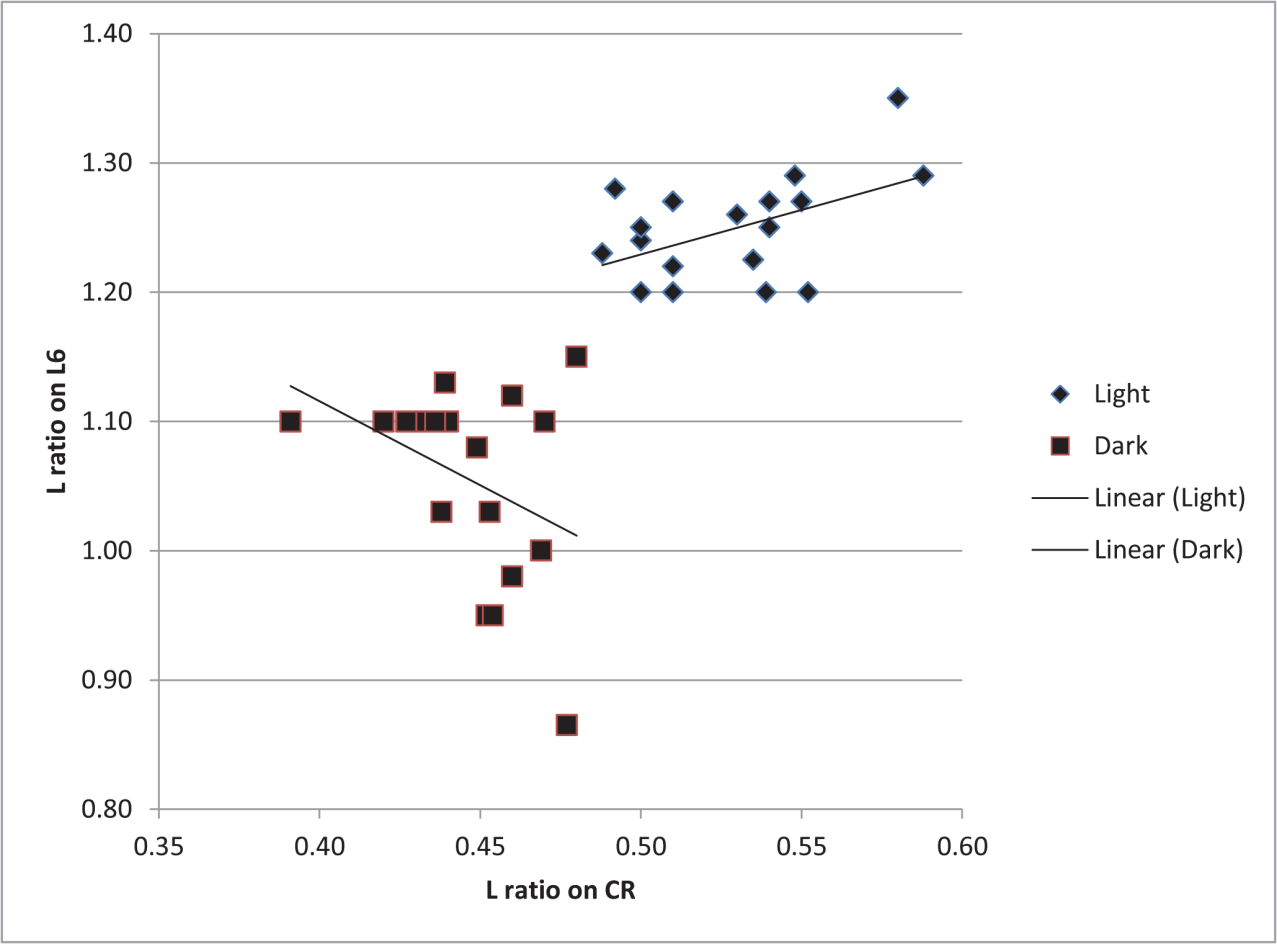
Correlation between morphometric data measured on the walking leg and uropodal ramus in two forms of *Physocypria biwaensis*. For morphometric abbreviations see Fig. 5.

The measurements taken from each examined specimen were used to calculate the following morphometric values: height/length ration (H/L); ratio between lengths of the anterior claw on the uropodal ramus and the ramus itself (AC/CR); ratio between lengths of the posterior claw and the ramus (PC/CR); ratio between lengths of anterior and posterior claws (PC/AC), and ratio between length of the claw on the walking leg and the second and third segments combined on the same leg (CL6/S). Average values and standard deviation were calculated for each measurement and the former is presented on a histogram (Fig. 6) and a t-Test assuming equal variances was used to test if the differences between mean values for the measured variables are statistically significant (S1 Table). Scatter plots were created for the following variables: shell length and height (Fig. 7), all morphometric data measured on the soft parts in the function of the shell length (Fig. 8), and one additional scatter plot showing the correlation between CL6/S and AC/CR (Fig. 9). S2 Table summarizes correlation indexes and 2-tailed p-values calculated for several morphometric data.

All obtained sequences were visualized using Finch TV version 1.4.0 (http://www.geospiza.com/Products/finchtv.shtml). BLAST [27] analyses of the GenBank database revealed that the obtained sequences were ostracod in origin and not contaminants. Each sequence was checked for the quality of signal and sites with possible low resolution, and corrected by comparing forward and reverse strands. Sequences were aligned in MEGA 6 [28] with ClustalW [29] using default paramaters. The majority of sequences were 660 bp long (the longest sequence was 672 bp, while the shortest was 408 bp). After the alignment each sequence was checked for potential stop codons with ORF finder on the NCBI Website (http://www.ncbi.nlm.nih.gov/projects/gorf/), using the invertebrate mitochondrial code. No stop codons were detected and sequences gave a translated polypeptide of approximately 216 amino acids, as expected for a functional *COI* gene. Before performing all subsequent calculations and analyses, most of the identical sequences were removed so that the final alignment contained 23 sequences: three belonging to *P*. *nipponica* and 10 each to the light and dark forms of *P*. *biwaensis*. The K2P model [30] was used to calculate the pair-wise distances between sequences (S3 Table). The sequences were divided into three groups (*P*. *nipponica*, *P*. *biwaensis* light form, and *P*. *biwaensis* dark form) and mean distance values between and within groups were also calculated using MEGA (S4 Table). The following phylogenetic analyses were performed: Maximum Likelihood (ML) using MEGA6, Bayesian Inference (BI) using MrBayes v3.3.3 [31, 32], and Maximum Parsimony (MP) using PAUP 4.0a136 [33], using the best fit evolutionary model for the former two. The BI analysis of *COI* data was carried out using default uninformative priors with four chains run simultaneously for five million generations in two independent runs, sampling trees every 500 generations. Of the four chains three were heated and one was cold, the temperature values (“Temp” command in MrBayes) was 0.1 (default option). Trees from each MrBayes run were combined and a burn-in of 1000 trees (10% of the total) was chosen, with a >50% posterior probability consensus tree constructed from the remaining trees. In the ML and MP analyses the bootstrap values [34] were calculated with 1000 pseudo-replicates. In the ML method I used partial deletion (95%), Nearest-Neighbor-Interchange (NNI) as the heuristic search method, and the initial tree was created automatically (Default-NJ/BioNJ), while for MP the heuristic search with tree-bisection-reconnection (TBR) branch swapping algorithm was used. For the best fit evolutionary model program jModelTest 2.1.6 [35, 36] was used with Akaike information criterion [37]. All trees were finally rooted with the three sequences of *P*. *nipponica*. Sequences are publicly available on GenBank (S5 Table).

## Results

### Morphological analysis

Comparison of the mean values of all morphometric data between the two forms indicates that all average values for the dark form are smaller than for the light form (Fig. 6) and the t-Test statistics (S1 Table) shows that these differences are highly significant. For example, the smallest measured light individual was 0.55 mm long while the largest was 0.62 mm (the 95% confidence interval for the light form lies between 0.58 mm and 0.59 mm). On the other hand, the length of the dark forms varies between 0.51 mm (minimum) and 0.58 (maximum) (with the 95% confidence interval between 0.52 mm and 0.53 mm) (Fig. 7). One measured dark form (DB, 79 m, F) was an obvious outlier in all taken measures, but it was not removed from the analysis in order to show the possible range of variability. There were no such clear outliers in the measured light forms.

It is to be expected that larger specimens also have comparatively longer appendages, but this would not affect the length ratios measured on the walking leg and the uropodal ramus. Scatter plot for these ratios as a function of the shell length (Fig. 8) shows that for most of the measurements dark animals (being smaller) have smaller values compared with light animals, but there is not a statistically significant correlation between the length of the shell and the soft parts’ measurements (S2 Table). The only exception is the correlation between the shell length and the ratio between posterior claw and the uropodal ramus in the dark form. In this case the p-value is 0.02, which is statistically significant and shows that the bigger the animal the longer is the posterior claw on the uropodal ramus. One morphometric measurement clearly stands out on the scatter plot (Fig. 8) and can be used to distinguish the two forms: the light form has a considerably longer claw on its walking leg (in comparison to the length of the two segments on the same leg) than the dark form. The smallest ratio in the light form is 1.2 mm and the longest 1.35 (95% confidence interval lies between 1.23 and 1.27); in the dark form the smallest measured ratio is 0.86 mm and the highest 1.15 mm (95% confidence interval lies between 1.00 and 1.1). These data suggest that there is no overlap between these values measured from the two forms.

A notable positive correlation was detected in the light form between the length ratio of the claw and segments on the walking leg and anterior claw and uropodal ramus. This correlation was statistically significant (p-value 0.03, see S2 Table). The same correlation in the dark form is, on the other hand, negative, but the p-value is above the level of confidence. Interestingly, there is a negative correlation between the walking leg measurements and the ratio between posterior claw and uropodal ramus in the dark form and here the p-value is 0.05, which is the threshold value.

### Molecular analyses

The highest pair-wise distance (23%; see S3 Table) is recorded between the *COI* sequences of *P*. *nipponica* and the light form of *P*. *biwaensis*, while the highest distance between the dark form and *P*. *nipponica* was 22%. K2P pairwise distances between the light and the dark form varied from 4% to 6%, with the average value of 5% (S4 Table). While the distances within the light form varied from 0 to 3% (the mean is 1.4%), in the dark form the variability was less pronounced (from 0 to 1%, with the mean value of 0.5%). The within group mean value for *P*. *nipponica* was 0.3%.

The total length of the alignment was 672 bp, of which 534bp were constant among haplotypes from the dark and light forms, 11 were variable characters and not parsimony informative, while 127 were parsimony informative. The MP analysis resulted in 5 equally parsimonious trees with a length of 165 steps, a consistency index of 0.91, a retention index of 0.97, and a composite index for parsimony informative sites of 0.89.

**Fig 10:**
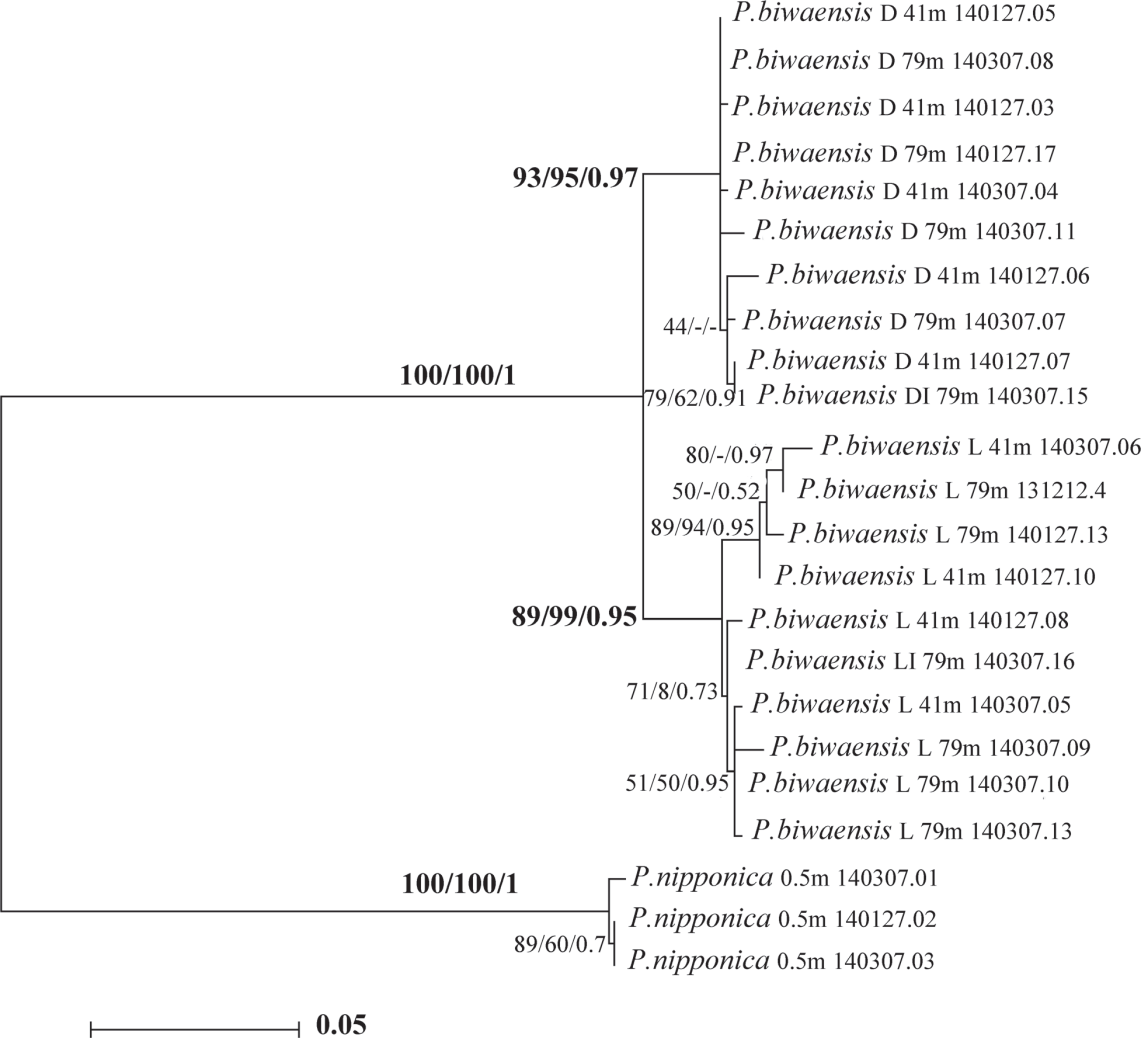
The ML cladogram of *Physocypria biwaensis* from Lake Biwa, based on *COI* sequences. Numbers above branches represent bootstrap values for the ML and MP analysis and BI 50% posterior probability values in the following order: ML/MP/BI. The tree is rooted with the *P*. *nipponica* clade and drawn to scale. L, light form; D, dark form.

The GTR model [38] with unequal rates among sites modeled using gamma distribution (GTR+G) was found to be the best fit evolutionary model based on the Akaike information criterion [37]. After five million generations runs in MrBayes, the final standard deviation of split frequencies had reduced to 0.003 and the potential scale reduction factor (PSRF) was ~1.0 for all parameters, suggesting convergence had been reached. Rates of chain mixing were between 20% and 40%. The ML analysis resulted in a tree with a very similar topology to that obtained by the BI method, and the bootstrap consensus tree from the MP analysis (Fig. 10). The two main branches contained the outgroup, and the ingroup had the highest possible bootstrap value. In addition, the branches representing the dark and light forms of *P*. *biwaensis* were supported with very high bootstrap values as well as a high posterior probability in all three analyses: for the dark form the values are 93 (ML), 95 (MP), and 0.97 (BI); for the light form these values are 89 (ML), 99 (MP), and 0.95 (BI). There is no evidence for a reciprocally monophyletic group of haplotypes each associated with specimens collected from 41 m and 79 m depths respectively. In fact, each of the two main haplotype groups contained a mixture of specimens from each of the collection depths. The intermediate forms (see Fig. 4) cluster with dark or light forms, corresponding to the observations noted below regarding the lateral outline, which is one of the major morphological distinguishing characters.

## Discussion

The most notable difference between the two forms is the shell shape; the dark form has a more rounded posterior margin than the light form. Although authors [20] observed specimens which are more or less elongated (most likely equivalent to what I refer to as more rounded or less rounded posterior margins), they also reported transitional forms. I could not observe any intermediate forms, and all specimens collected fitted into one of the two groups: either more rounded (corresponding to the dark form), or globular (corresponding to the light form).

Morphometric analysis of the soft parts and the shell of the light and dark forms of *Physocypria biwaensis* identified two important morphological differences: the light form is on average bigger (0.59 mm) than the dark form (0.53 mm), and the terminal claw on the walking leg is longer in comparison to the length of the two segments in the light form (mean value 1.25) than in the dark one (1.1). The light form also tends to be slightly lighter in its pigmentation in the deeper parts of the lake. Depigmentation with an increase of darkness (in subterranean waters, for example) is well documented in ostracods [39, 40], as is the loss of eye pigmentation and other morphological adaptations. I have not detected any difference in eye pigmentation between populations from the 41 m and 79 m depths, as well as any difference in the morphology of soft parts.

It is worth noting that the light form is more numerous in the deeper parts of the lake (500+ specimens) both in the comparison to its population from the shallower part (30+ specimens) and the dark population from the same depth (20+ specimens). All light traps were of the same size, so the samples were quantitative. The darker form is slightly more numerous in the shallower parts of the lake (50+ specimens). This may indicate that the light form dominates at the lower depths, but this would have to be tested with a larger sampling effort. Another discrepancy between the light and dark forms is that the males are more abundant sex in the latter at both depths, while the females are more abundant sex for the light form. Over dominance of males of some marine myodocopid ostracods in light traps can be explained by the fact that males often have better developed eyes with more omatidia, or females lack eyes altogether (see [41] diagnosis for different myodocopid taxonomic units). In some cases, light traps from marine environments have failed to catch any females (personal observation). However, this is not the case with the freshwater ostracods, and in particular *P*. *biwaensis*, and it is not an explanation for the dominance of females of the light form in the traps. Although no morphological adaptations to the deep water are evident for the two forms, the correlation between morphometric measurements on the walking leg and the uropodal ramus, is statistically significant, and may hint at some kind of adaptation to the deeper environment by the light form. This morphometric analysis indicated that the longer the claw on the walking leg the longer is the anterior claw on the uropodal ramus. On the other hand, in the dark form there is a negative correlation in these measurements, i.e. the longer the claw on the walking leg the shorter the claw on the uropodal ramus. This negative correlation is not, however, statistically significant, but should be checked on a larger series of specimens. The importance of this trend is hard to explain, since these animals have long swimming setae on both antennae, so they actively swim as well as walk. Elongation of claws and segments on ostracod appendages is well documented for some subterranean taxa [39], but this can be explained by the fact that these ostracods cannot swim, so their mobility depends on the development of other parts of the locomotory apparatus.

Although one sample came from the littoral zone of Lake Biwa none of the forms of *P*. *biwaensis* in the current study were collected from there. Okubo [25] described the species from the littoral zone of the lake and Smith & Janz [20] stated that the species occurred from 0.1 to 87.4 m in depth. This finding requires further checking given the morphometric and molecular results presented here.

All three methods used for the phylogenetic reconstruction revealed similar topologies (Fig. 10): *P*. *nipponica* is a distant relative of *P*. *biwaensis*, with average pairwise K2P distances of 22% between the former and the dark form, and 23% between it and the light form. The *P*. *biwaensis* clade was clearly divided into two well supported clades, each comprising either the light or the dark form specimens. Specimens with an intermediate pigmentation, which were very rare (see above), clustered within their appropriate form based on the shell outline. The average pairwise distance between the dark and the light form clades was 5%, which is relatively low compared to most recognized crustacean species [8]. So far, the lowest *COI* divergence values between two morphologically recognizable ostracod species is 6.1% (p-distance) or 6.7% (Tamura 3 parameter), and that was recorded between *Bennelongia ivanae* Martens, Halse & Schön, 2013 and *B*. *sp*. *nov*. F2 [7]. In that paper, the authors also reported even greater distances (14%) between some populations of *B*. *timmsi* Martens, Halse & Schön, 2013, but could not find any morphological differences. The K/θ method [15] used by Schön *et al*. [6] to study cryptic speciation in Darwinulidae ostracods supported the existence of seven cryptic species within the almost cosmopolitan *Penthesilenula brasiliensis* (Pinto & Kotzian, 1961), among which the smallest *COI* pairwise distance (between two Brazilian populations) was only 4.4%.

The current state of ostracod taxonomy is such that these small genetic distances between the two Lake Biwa forms of *P*. *biwaensis* would not be enough alone to indicate the presence of discrete speciation if these were allopatric populations. Higashi *et al*. [42] studied several populations of *Microloxoconcha dimorpha* Higashi *et al*., 2011, an interstitial ostracod with high morphological variability. Phylogenetic trees constructed based on *COI* sequences did not support the presence of separate species for the different morphological forms, while the genetic distance ranged from 0.2 to 0.8% between geographically close populations and slightly more than 6% between allopatric populations. They concluded that this higher genetic distance is a result of a slow (non-existent) genetic flow, but it does not support separate species. The authors, however, have not conducted any interbreeding experiments, or collected the same species in areas laying in-between the two disjunct populations. In the case of *P*. *biwaensis* the two forms are not geographically separated and they seem to freely migrate between the different depths, given that exactly the same *COI* haplotypes can be found at 41 m and 79 m depths. Birky [15] expressed the possibility that a high K/θ ratio between two potentially cryptic species may result in migration of females between the populations, suggesting that the differences in the *COI* are only apparent and that the interbreeding does persist. In the case of *P*. *biwaensis* the genetic distance and phylogenetic grouping of individuals, is associated with distinct morphological differences in sympatry, supporting their distinction as separate species. However, it should be noted that the morphological characters traditionally used to separate ostracod species, such as male sexual characteristics, are indistinguishable between the two forms, and are also highly variable.

Low divergence rates suggest that the two species of *P*. *biwaensis* have been separated relatively recently. Although I have not used molecular clock analyses, if the most commonly used divergence rates for mitochondrial *COI* of 1.4% and 2.6% per million years in crustaceans [43, 44] are considered, the two forms of *P*. *biwaensis* may have separated between one and two million years ago. This separation may coincide with the origin of the ancient Lake Biwa [21]. It is possible that one of the species evolved in some parts of the ancient Lake Biwa and colonized deeper regions as it evolved, probably following the temperature gradient, while the other evolved elsewhere and recently started colonizing it. It is more likely that the light form evolved in the lake because it seems to be better adapted to the deep (with the reduction of its pigmentation), and it is more dominant there than the dark form. The light form also has a much higher intrapopulation genetic variability (1%-3%) which may indicate either its longer evolution in the lake or several independent colonizations.

The results of this study support the presence of two distinct species within the *P*. *biwaensis* complex in Lake Biwa. Judging by the original description [25], the current name is associated with the dark form, from the current study, because it has a more rounded posterior margin of the carapace and shorter claws on the uropodal ramus. Therefore, the light form represents an undescribed new species. The taxonomic description of this species will follow after the type material of *P*. *biwaensis* has been examined.

## Supporting Information

S1 Table The p-values (at α = 0.01) for a t-Test assuming equal variances for the differences in mean values of measured variables between the light and dark forms of *P*. *biwaensis*. (DOCX)

S2 Table Correlation coefficient and its 2-tailed p-values (between brackets, α = 0.05) between different measurements in light (L) and dark (D) forms of *Physocypria biwaensis*, (DOCX)

S3 Table Pairwise (K2P) *COI* sequence distances. (XLS)

S4 Table Between and within group mean sequence distance values (K2P model, pairwise deletion). (DOCX)

S5 Table GenBank Accession Numbers. (DOCX)
