# Supplementary material for: Barcoding of Ancient Lake Ostracods (Crustacea) Reveals Cryptic Speciation with Extremely Low Distances
Source: PLoS One. 2015 Mar 26;10(3):e0121133. doi: 10.1371/journal.pone.0121133 (PMC4374928; doi:10.1371/journal.pone.0121133)
Supplement: S1 Table — (DOCX) [file pone.0121133.s001.docx]

S1 Table. The p-values (at α=0.01) for the t-Test assuming equal variances for the differences in mean values of measured variables between the light and dark forms of *P. biwaensis*

|  | p-value (α=0.01) |
| --- | --- |
| L | 2.02202E-12 |
| H | 6.88E-15 |
| AC/UR | 5.40E-11 |
| PC/UR | 1.46E-11 |
| PC/AC | 0.00E+00 |
| CL6/L | 2.13E-11 |
