# Supplementary material for: Barcoding of Ancient Lake Ostracods (Crustacea) Reveals Cryptic Speciation with Extremely Low Distances
Source: PLoS One. 2015 Mar 26;10(3):e0121133. doi: 10.1371/journal.pone.0121133 (PMC4374928; doi:10.1371/journal.pone.0121133)
Supplement: S2 Table — (DOCX) [file pone.0121133.s002.docx]

S2 Table. Correlation coefficient and its 2-tailed p-values (between brackets, α=0.05) between different measurements in light (L) and dark (D) forms of *Physocypria biwaensis*

|  | L | D |
| --- | --- | --- |
| L & H | 0.99 (0.00) | 0.86 (0.00) |
| L & AC/UR | 0.267 (0.27) | 0.05 (0.84) |
| L & PC/UR | 0.41 (0.08) | 0.54 (0.02) |
| L & PC/AC | 0.06 (0.80) | 0.42 (0.08) |
| L & CL6/AC | -0.04 (0.87) | 0.07 (0.78) |
| AC/UR & PC/UR | 0.81 (0.00) | 0.48 (0.04) |
| CL6/S & AC/UR | 0.49 (0.03) | -0.37 (0.13) |
| CL6/S & PC/UR | 0.18 (0.46) | -0.47 (0.05) |
