# Supplementary material for: Barcoding of Ancient Lake Ostracods (Crustacea) Reveals Cryptic Speciation with Extremely Low Distances
Source: PLoS One. 2015 Mar 26;10(3):e0121133. doi: 10.1371/journal.pone.0121133 (PMC4374928; doi:10.1371/journal.pone.0121133)
Supplement: S4 Table — (DOCX) [file pone.0121133.s004.docx]

S4 Table. Between and within group mean sequence distance values (K2P model, pairwise deletion)

|  | *P. biwaensis* (D) | *P. biwaensis* (L) | *P. nipponica* |
| --- | --- | --- | --- |
| *P. biwaensis* (D) | 0.005 | - | - |
| *P. biwaensis* (L) | 0.05 | 0.014 | - |
| *P. nipponica* | 0.22 | 0.23 | 0.003 |
