# Supplementary material for: Barcoding of Ancient Lake Ostracods (Crustacea) Reveals Cryptic Speciation with Extremely Low Distances
Source: PLoS One. 2015 Mar 26;10(3):e0121133. doi: 10.1371/journal.pone.0121133 (PMC4374928; doi:10.1371/journal.pone.0121133)
Supplement: S5 Table — (DOCX) [file pone.0121133.s005.docx]

S5 Table. GenBank accession numbers for the *COI* sequences used in the phylogenetic analysis

| Species | Label on the phylogenetic tree (Fig. 10) | Accession Number |
| --- | --- | --- |
| *Physocypria biwaensis* | *P. biwaensis* D 41m 140127.03 | KP842892 |
| *Physocypria biwaensis* | *P. biwaensis* D 41m 140127.05 | KP842893 |
| *Physocypria biwaensis* | *P. biwaensis* D 41m 140127.06 | KP842894 |
| *Physocypria biwaensis* | *P. biwaensis* D 41m 140127.07 | KP842895 |
| *Physocypria biwaensis* | *P. biwaensis* D 41m 140307.04 | KP842896 |
| *Physocypria biwaensis* | *P. biwaensis* D 79m 140127.17 | KP842897 |
| *Physocypria biwaensis* | *P. biwaensis* D 79m 140307.07 | KP842898 |
| *Physocypria biwaensis* | *P. biwaensis* D 79m 140307.11 | KP842899 |
| *Physocypria biwaensis* | *P. biwaensis* D 79m 140307.08 | KP842900 |
| *Physocypria biwaensis* | *P. biwaensis* DI 79m 140307.15 | KP842901 |
| *Physocypria biwaensis* | *P. biwaensis* L 41m 140127.08 | KP842902 |
| *Physocypria biwaensis* | *P. biwaensis* L 41m 140127.10 | KP842903 |
| *Physocypria biwaensis* | *P. biwaensis* L 41m 140307.05 | KP842904 |
| *Physocypria biwaensis* | *P. biwaensis* L 41m 140307.06 | KP842905 |
| *Physocypria biwaensis* | *P. biwaensis* L 79m 131212.04 | KP842906 |
| *Physocypria biwaensis* | *P. biwaensis* L 79m 140127.13 | KP842907 |
| *Physocypria biwaensis* | *P. biwaensis* L 79m 140307.09 | KP842908 |
| *Physocypria biwaensis* | *P. biwaensis* L 79m 140307.10 | KP842909 |
| *Physocypria biwaensis* | *P. biwaensis* L 79m 140307.13 | KP842910 |
| *Physocypria biwaensis* | *P. biwaensis* LI 79m 140307.16 | KP842911 |
| *Physocypria nipponica* | *P. nipponica* 0.5m 140127.02 | KP842912 |
| *Physocypria nipponica* | *P. nipponica* 0.5 m 140307.03 | KP842913 |
| *Physocypria nipponica* | *P. nipponica* 0.5m 140307.01 | KP842914 |
